# Coprogen B from *Talaromyces marneffei ΔsreA*: Rapid Iron Chelation and Favorable Partitioning to Deferoxamine

**DOI:** 10.3390/ijms262311281

**Published:** 2025-11-21

**Authors:** Bishant Pokharel, Wachiraporn Tipsuwan, Monsicha Pongpom, Teera Chewonarin, Pimpisid Koonyosying, Agostino Cilibrizzi, Somdet Srichairatanakool

**Affiliations:** 1 Department of Biochemistry, Faculty of Medicine, Chiang Mai University, Chiang Mai 50200, Thailand; bishant_p@cmu.ac.th (B.P.); teera.c@cmu.ac.th (T.C.); pimpisid.k@cmu.ac.th (P.K.); 2 Division of Biochemistry, School of Medical Science, University of Phayao, Phayao 56000, Thailand; wachiraporn.ti@up.ac.th; 3 Department of Microbiology, Faculty of Medicine, Chiang Mai University, Chiang Mai 50200, Thailand; monsicha.p@cmu.ac.th; 4 Institute of Pharmaceutical Science, King’s College London, London SE1 9NH, UK; agostino.cilibrizzi@kcl.ac.uk

**Keywords:** siderophore, coprogen, talaromyces, iron, chelator, partition, cLogP

## Abstract

Iron (Fe) chelators are used to treat iron-overloaded disorders, metal detoxification, radionuclides, and molecular imaging; however, they can cause side effects. In this study, we identified and characterized Coprogen B (CPGB), a hexadentate trihydroxamate siderophore secreted by the opportunistic dimorphic fungus *Talaromyces marneffei* and compared its properties with deferoxamine (DFO). Siderophore production was enriched from a *ΔsreA* strain and purified via Amberlite XAD2 and Sephadex LH20 chromatography, followed by reverse-phase HPLC. Active fractions were confirmed by Ultraviolet–Visible (UV–Vis) spectral fingerprints (≈230 nm) for hydroxamate, with a band at 430–450 nm upon Fe(III) complexation, as well as by chrome azurol A assay, Nuclear Magnetic Resonane (NMR) spectroscopy, High-Performance Liquid Chromatography–Mass Spectrometry (HPLC-MS), and Matrix-Assisted Laser Desorption/Ionization–Time-of-Flight Mass Spectrometry (MALDI-TOF-MS). CPGB exhibited strong molar absorptivity and rapid, concentration-dependent chelation of Fe(III), yielding a sustained binding profile that matched or exceeded that of DFO over time. In determining *n*-octanol/water partitioning for CPGB and DFO (230 nm) and their Fe(III) complexes, the partitioning (P) assay revealed that CPGB was moderately hydrophilic (P = 0.505 ± 0.063; cLogP = −0.299 ± 0.053), while DFO was strongly hydrophilic (P = 0.098 ± 0.005; cLogP = −1.010 ± 0.022). Fe(III) complexation reduced lipophilicity: CPGB–Fe partitioned ~30–35% into octanol, while DFO–Fe complex partitioned ~7–8%, remaining largely aqueous. Overall, this outcome potentially suggested improved clearance in vivo. These data nominate CPGB as a promising alternative to existing iron chelators. The siderophore exhibited greater lipophilicity, emphasizing better passive membrane permeability than DFO, while siderophore–Fe(III) binding indicated increased biases toward the aqueous phase. Future in vivo studies are warranted to confirm its pharmacokinetics, safety, and therapeutic efficacy.

## 1. Introduction

Iron is a crucial bioelement for living organisms. It plays a pivotal role in numerous cellular mechanisms, including DNA metabolism, protein function, synthesis of fatty acids, and other biochemical reactions. Belonging to the group of transition metals, iron exists in two oxidation states: ferric (Fe^3+^) and ferrous (Fe^2+^). Typically, Fe^3+^ is poorly soluble under aerobic conditions, which renders it less available to biological systems. To overcome this limitation, microorganisms have intrinsically evolved as mechanisms to acquire Fe^3+^ by secreting high-affinity iron-chelating molecules known as siderophores. Siderophores are small molecules synthesized by bacterial and fungal species that possess the ability to chelate metals [1]. They specifically capture Fe^3+^ and transfer it to microbial cells via specific receptors. Their primary role is to deliver iron to microbes for their survival and growth; nevertheless, they have also attracted significant attention due to their potential benefits in clinical settings. Examples include, but are not limited to, the introduction of the Trojan-horse strategy, as well as diagnostic assays and their benefits, to chelate iron in patients facing iron overloading.

In the Trojan-horse strategy, a complex between an antimicrobial drug and a siderophore facilitates the transport of antibiotics into pathogens by mimicking a siderophore–iron complex, which the pathogens require to satisfy their hunger for iron. For instance, the spermidine-based biscatechol–cephalsporin complex and deferoxamine (DFO)–nalidixic acid complex have been reported to restrict the growth rate of pathogenic bacteria [2]. Furthermore, siderophores have proven to be helpful in diagnosing certain infectious diseases, such as through radiolabeling Gallium (III) as a [^67/68^Ga]-DFO complex, and when they have been used as an imaging tool to precisely locate *Staphylococcus aureus* infection in mice [3]. In the treatment of diseases characterized by iron overload, DFO, a hexadentate siderophore and commercially available FDA-approved drug [4], is parenterally administered for single, as well as combination, therapy along with other chelators. The drug has been shown to alleviate labile plasma iron and non-transferrin bound iron, consequently ameliorating oxidative stress in transfusion-dependent thalassemia patients [5,6,7]. Due to the adverse effects of DFO [8], oral iron chelators, such as deferasirox (DFX) and deferiprone (DFP), were developed to cure iron overloading. Nonetheless, persistent safety concerns signify the urgent desire to discover novel, potent, and safer iron chelators that can assure the long-term safety of patients.

Among fungal species, *Talaromyces marneffei* naturally synthesizes a powerful extracellular siderophore named “Coprogen-B (CPGB)” [9,10]. CPGB production is regulated by two key transcriptional factors, HapX and siderophore-regulatory element (SreA), which act as genetic switches to create a feedback loop [11,12]. When the fungus requires iron, a factor called HapX is activated, leading to increased siderophore production. Conversely, when iron levels are high, SreA functions as an “off switch,” shutting down overproduction to prevent toxic iron accumulation. This significant on/off mechanism provides researchers with clear and simple insight into the mechanism that can be used to develop a siderophore factory by removing the “off switch”, i.e., SreA. By deleting the *sreA* gene, the fungus would not receive the stop signal for siderophore production, leading to a massive production of the desired compound. Moreover, a recent study has revealed the transcriptional activation of some genes involved in siderophore production pathways [9].

Techniques such as solvent extraction (e.g., benzyl alcohol and ethyl acetate), preparative and analytical liquid chromatography (e.g., adsorption, ion-exchange, size exclusion, and affinity) [13], and electrophoresis are commonly used for the purification of microbial siderophores and synthetic chelators. Detection and characterization may be performed using specific colorimetry methods such as Arnow’s, Csaky’s and chrome azurol S (CAS), nuclear magnetic resonance (NMR) spectroscopy, Fourier-Transform infrared (FT-IR), ultraviolet/visible (UV/Vis) spectrophotometry, X-ray diffraction (XRD), and mass spectrometry (MS), which may also involve fast atom bombardment (FAB), matrix-assisted laser desorption/ionization (MALDI), or quadrupole time-of-flight (QTOF) [13,14,15].

Hence, based on these concepts and other recent discoveries, this current study was designed to apply the *sreA* deletion technique to *T. marneffei*. The newly engineered strain was subjected to identification, purification, and analysis by various initial testing methods to confirm its potential output in terms of iron chelation and lipophilicity.

## 2. Results

### 2.1. Assessment of Siderophore in T. marneffei Culture Supernatant

**Figure 1:**
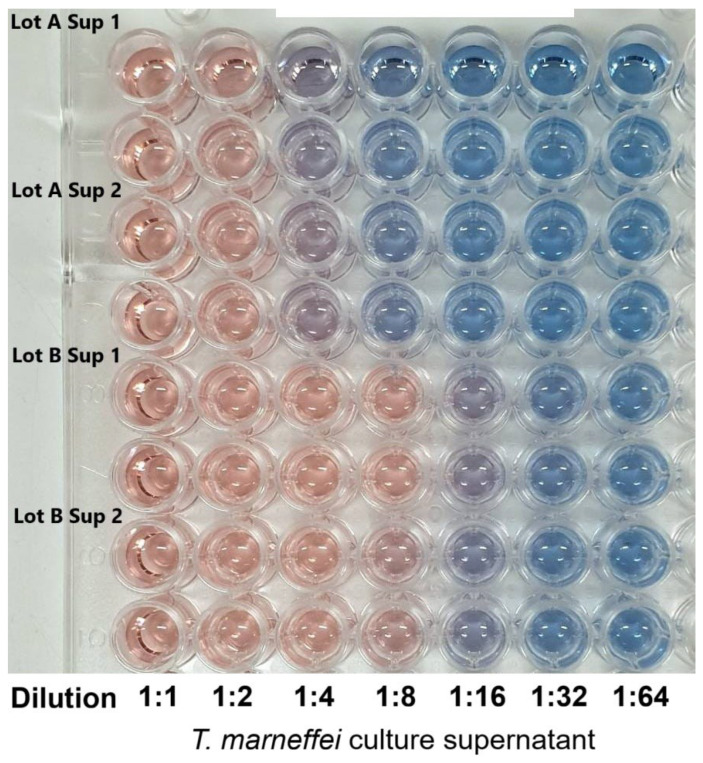
CAS-based detection of siderophores in *T. marneffei* culture supernatant diluted from 1:1 to 1:64.

The supernatant obtained from *T. marneffei* culture tested positive for siderophore production using CAS assay. The assay indicated the presence of siderophore in the fungal culture. Moreover, the culture was found to be strongly positive at an up to 8-fold dilution, while appreciable siderophore levels were detected even at a 16-fold serial dilution (Figure 1).

### 2.2. Identification of Positive Fractions Post Chromatogaphy

**Figure 2:**
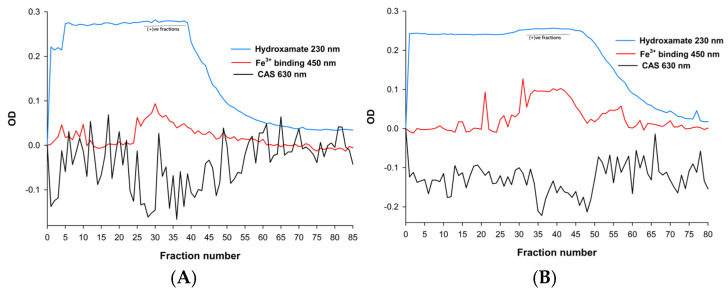
Profiles of Amberlite XAD-2 (**A**) and Sephadex LH-20 (**B**) column chromatographic fractions obtained from *T. marneffei* culture medium monitored by OD measurements at 230 and 450 nm, and CAS assay.

Following separation using Amberlite XAD-2 resin adsorption and Sephadex LH-20 resin gel permeation chromatography, the positive fractions were identified through a combination of spectral analysis, an assessment of iron-binding capacity, and CAS assay. Fractions that tested positive across all three criteria were then pooled for subsequent experiments. These measurements facilitated the identification of fractions containing siderophores with iron-binding activity (450 nm), CAS activity (630 nm), and the characteristic hydroxamate-type siderophore OD values at 230 nm (Figure 2A,B). Fractions 26–39 from Amberlite XAD-2 and fractions 35–48 from Sephadex LH-20 tested positive for siderophore activity.

### 2.3. Characterization of Crude Siderophore

**Figure 3:**
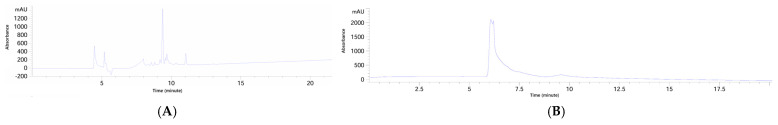

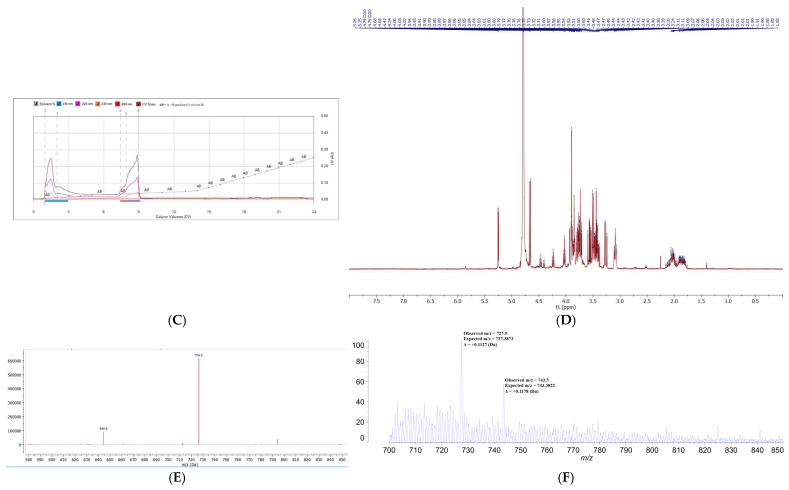
HPLC-DAD (**A**), preparative HPLC (**B**), flash chromatogram (**C**), NMR spectra (**D**), ESI-MS (**E**), and MALDI-TOF-MS (**F**) profiles for OD 230 and 450 nm, and CAS-positive fractions.

Results for the positive fractions obtained from Sephadex LH-20 gel permeation followed by analytical HPLC-DAD demonstrated that the compounds associated with an OD value of 230 nm were indicative of hydroxamate-type siderophores. They were predominantly separated at two distinct retention times. Separation occurred at approximate retention times (T_R_) of 5 and 10 min (Figure 3A). These two peaks suggested the presence of compounds with differing levels of hydrophobicity, but which exhibited the same OD peaks. Flash chromatography of the crude sample yielded results consistent with the analytical HPLC (Figure 3C), revealing two sets of compounds with maximal absorption at 230 nm: one eluting between the first- and third-column fractions, and the other between the eighth- and ninth-column fractions. In HPLC, a gradual increase in solvent B (0.1% formic acid (FA) in acetonitrile) against solvent A (0.1% formic acid, FA in deionized (DI) water) resulted in elution at approximately 5 min and again at 10 min. The second peak was presumed to represent CPGB, as it eluted under conditions indicative of a less polar compound. In contrast, flash chromatography employed a reversed solvent system, with solvent B (DI water) gradually increasing against solvent A (acetonitrile), thereby producing two peaks. Herein, the first peak corresponded to CPGB and aligned with the second HPLC peak.

NMR spectroscopy was performed on the crude sample, producing spectra results (Figure 3D) comparable to those of coprogen previously reported by Huang et al. [16]. However, the presence of additional signals indicated the crude nature of the sample. While CPGB was present, the spectra confirmed the coexistence of a relatively low level of impurities, necessitating further purification. Similar findings obtained from MALDI-TOF-MS analysis with sinapinic acid (SA) matrix showed the presence of a peak at a mass-to-charge (*m*/*z*) value of 727.5 (Figure 3F), which was indicative of CPGB (MW = 726.8). The two peaks recorded at *m*/*z* 727.5 and *m*/*z* 743.5 correspond to the [M+H]^+^ and a possible hydroxylated species, respectively (Figure 3F). MALDI-TOF-MS was also performed using 2,5-dihydroxybenzoic acid (DHB) and α-cyano-4-hydroxycinnamic acid (CHCA) as matrices, yielding comparable results 

### 2.4. Separation of Coprogen of Interest

**Figure 4:**
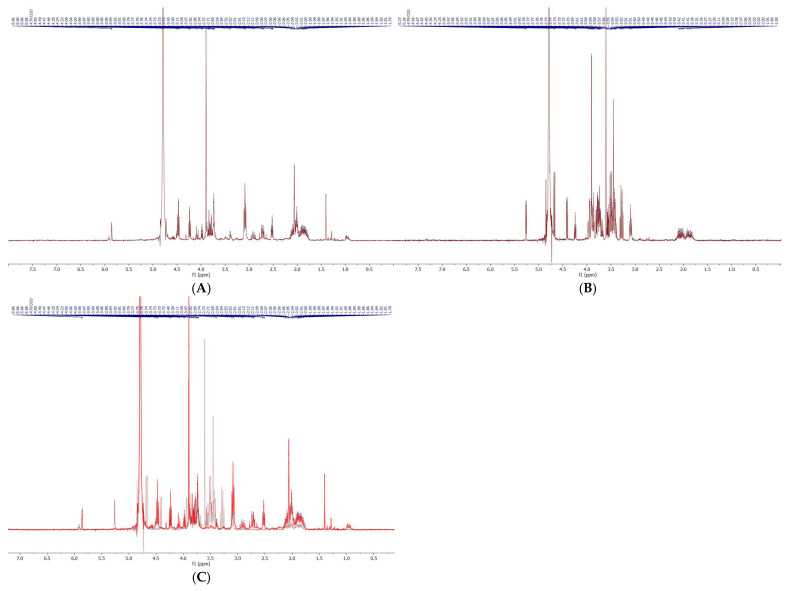
^1^ H-NMR spectra of fraction 1 (**A**) and fraction 2 (**B**), along with their overlaid spectra (**C**), collected from CombiFlash chromatography.

Fractions corresponding to the first peak at a specific retention time (T_R_ of 1–2 min) and the second peak (T_R_ of 8–9 min) obtained from flash chromatography were collected, pooled, and subsequently analyzed by NMR spectroscopy. Freeze-dried samples were dissolved in deuterium oxide for measurement. The first peak produced spectra identical to CPGB (Figure 4A), corroborating the findings of Huang et al. [16]. Conversely, the second peak yielded entirely distinct spectra (Figure 4B), confirming that this species was not CPGB.

### 2.5. Iron-Binding Effects of Siderophores

#### 2.5.1. Spectral Analysis

**Figure 5:**
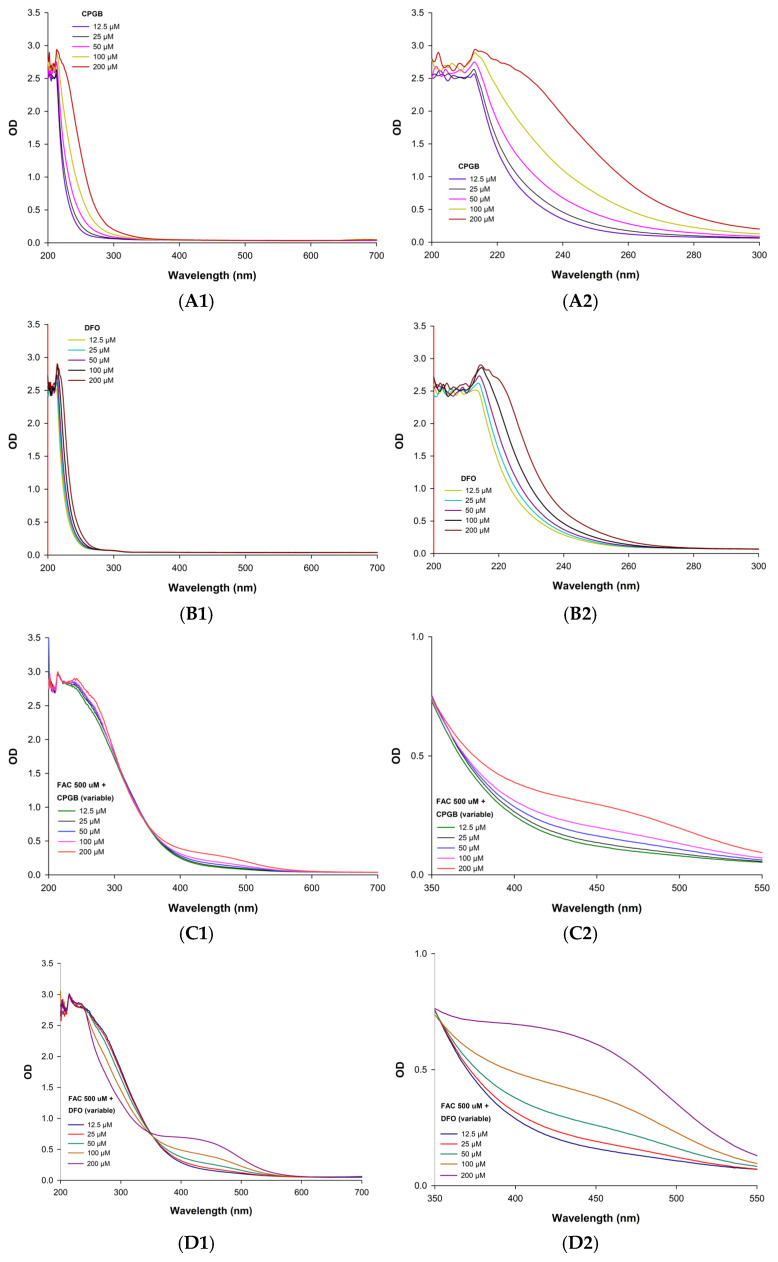
UV-Vis spectra of free CPGB (**A1**), DFO (**B1**), CPGB-Fe3+ (**C1**), and DFO-Fe3+ (**D1**) complexes, along with the corresponding zoomed-in spectral analysis of the CPGB (**A2**), DFO (**B2**), CPGB-Fe^3+^ (**C2**), and DFO-Fe^3+^ (**D2**) complexes.

CPGB demonstrated a hydroxamate-type absorbance peak near 230 nm with a concentration-dependent shoulder extending from 230 nm to 280 nm, which was consistent with a δ-lactone structural feature (Figure 5A), while DFO exhibited a similar 230 nm peak without the shoulder (Figure 5B). At low concentrations (≤50 µM), the spectra of CPGB and DFO demonstrated highly comparable profiles, which would be consistent with their shared hydroxamate-type coordination chemistry. Nonetheless, at higher concentrations (100 µM and 200 µM), the spectral differences became pronounced, notably in peak intensity and shoulder broadening values between 230 and 280 nm for CPGB. This variation could be attributed to the changing siderophore-to-iron stoichiometry. Titrations of the CPGB and DFO with 300 μM FAC pH 7.0 solution exhibited concentration-dependent Fe(III) binding for CPGB (Figure 5C) and DFO (Figure 5D). At low concentrations, the ratio of CPGB or DFO to 500 μM FAC was approximately 1:40 for the CPGB-Fe(III) and DFO-Fe(III) complexes, indicating that iron would be in large excess and both siderophores were undersaturated, thereby minimizing any differences in coordination behavior. At higher ligand concentrations (100 µM, a ratio of 1:5; 200 µM, a ratio of 1:2.5), the system approaches equimolar conditions, allowing distinct complexation equilibria to emerge. These conditions accentuate CPGB’s additional electronic transitions associated with its δ-lactone ring, leading to the more complex spectral envelope observed.

#### 2.5.2. Kinetic Iron Binding to Siderophores

**Figure 6:**
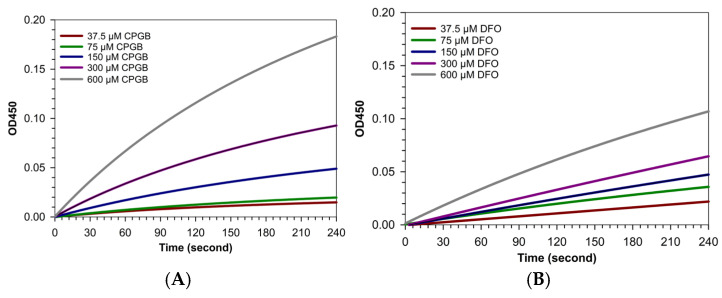
Time-course iron-binding activity of CPGB (**A**) and DFO (**B**). Data obtained from three separate experiments are shown in mean ± SD values.

**Figure 7:**
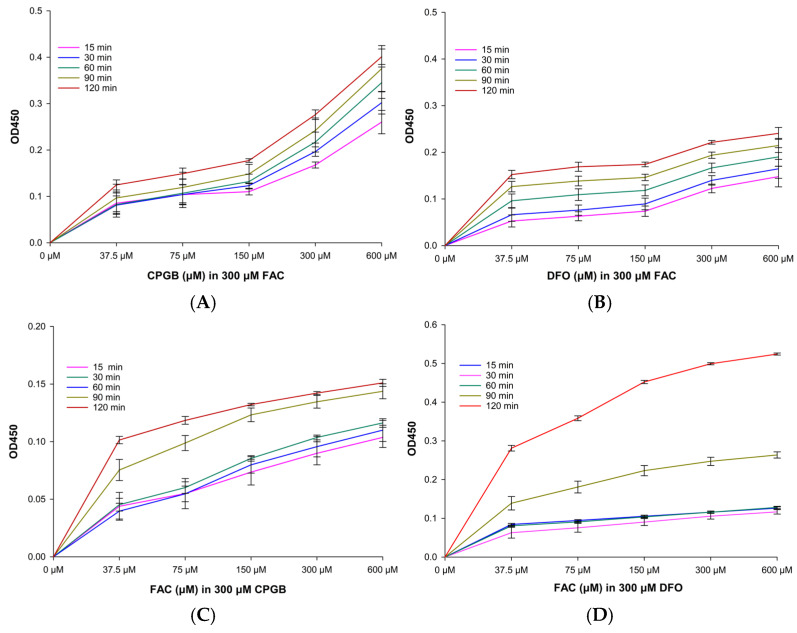
Kinetic iron-binding activity of CPGB (**A**,**C**) and DFO (**B**,**D**). Data obtained from three separate experiments are shown in mean ± SD values.

The iron-binding activity of DFO appeared to be immediate, as spectral analysis was performed directly after its addition to FAC (Figure 6A,B). To further examine the kinetics, a study was conducted wherein 300 µM FAC in 100 mM MOPS buffer was treated with various concentrations of CPGB and DFO. The results indicated that CPGB exhibited a strong but more gradual iron-binding profile when compared with DFO. Notably, over time, CPGB demonstrated greater binding capacity than DFO (Figure 7A–D).

Time-dependent assays of iron–siderophore complex formation revealed that CPGB displayed comparable, and under certain conditions, superior iron-binding activity relative to DFO. These findings suggest that CPGB possesses stronger chelating potential and a higher affinity for iron than DFO. While DFO exhibited rapid complex formation, CPGB showed a more gradual yet sustained binding profile. Importantly, at later time points, CPGB consistently surpassed DFO in binding capacity.

### 2.6. Partition Coefficient Assessment

**Figure 8:**
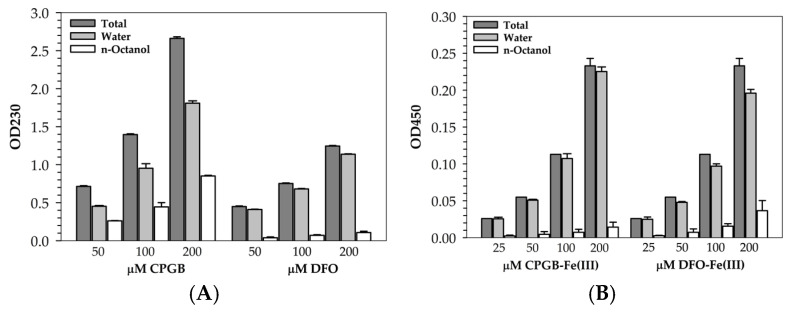
Partitioning CPGB and DFO per se (**A**), CPGB-Fe(III) and DFO-Fe(III) complexes (**B**) in water/*n*-octanol bi-phase. Data obtained from three separate experiments are shown in mean ± SD values.

The results showed measurements of OD values for CPGB and DFO (Figure 8A) and their iron complex (Figure 8B) at different concentrations in the aqueous MOPS phase before and after adding *n*-octanol, as well as in the *n*-octanol phase. The OD value at 230 nm of CPGB decreased after being partitioned into octanol as compared to before, indicating that CPGB was distributed in the aqueous and octanol phases. Similarly, OD values after octanol addition and in the octanol phase remained very close to the aqueous values, indicating DFO had lower partitioning into octanol than CPGB and remained mostly in the aqueous phase. The results suggest that CPGB had higher hydrophobicity/lipophilicity than DFO. When CPGB bound Fe(III), the complex became more hydrophobic than free iron but less polar than free CPGB, for which approximately 30–35% of the complex was partitioned into the octanol. The partitioning data (OD decreased after octanol addition) suggests that the iron complex retains moderate hydrophobicity and was distributed partly into nonpolar environments. The result implies that CPGB–Fe could cross lipid barriers more readily, potentially mimicking bacterial siderophores designed for membrane transport. DFO formed a very stable hexadentate complex with Fe(III) called ferrioxamine (FO), which is characterized as highly charged, hydrophilic, and bulky. Only 7–8% DFO-Fe(III) complex was partitioned into octanol, confirming that the iron complex remained in water with virtually no lipophilicity.

The formation of CPGB- and DFO-bound Fe(III) complexes modestly influenced the distribution of these two siderophores between the aqueous and organic phases. As is shown in Figure 8, both free siderophores were highly hydrophilic and remained largely in the aqueous phase. Upon complexation with Fe(III), minor shifts toward the organic phase were observed, particularly for CPGB-Fe(III), suggesting subtle alterations in solvation or molecular packing. Nevertheless, the overall partitioning ratios demonstrated that neither CPGB-Fe(III) nor DFO-Fe(III) were substantially soluble in *n*-octanol, and these complexes were unlikely to efficiently traverse lipid barriers under physiological conditions. The slightly higher apparent partitioning of CPGB-Fe(III) may instead reflect localized interactions at the water–octanol interface rather than true lipid solubility. Collectively, these results support the contention that CPGB maintains strong Fe(III) chelation that is comparable to DFO, but without significantly increasing its lipophilic transport potential.

**Figure 9:**
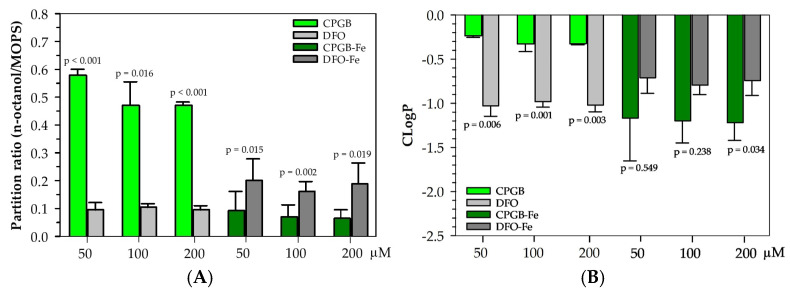
Partitioning (**A**) and cLogP (**B**) values of CPGB vs. DFO and CPGB-Fe vs. DFO-Fe complex in *n*-octanol at 50–200 µM. Data represent mean ± SD (n = 3) values.

Cooperatively, the partitioning and cLogP values shown in Figure 9 further support the hydrophilic nature of both CPGB and DFO, as reflected by their low cLogP values. Across the 50–200 µM concentration range, both ligands and their corresponding Fe(III) complexes exhibited cLogP values well below zero, indicating a strong preference for the aqueous phase. A slight increase in apparent cLogP (less negative value) was observed for CPGB-Fe(III) when compared with DFO-Fe(III), which was consistent with the subtle shift seen in biphasic partitioning (Figure 8). However, this change remained within a narrow range and did not imply a true lipophilic characteristic. Instead, it likely arose from small alterations in the hydration shell structure or weak interfacial associations of the δ-lactone-modified complex. Taken together, these data reinforce the contention that CPGB remains largely hydrophilic even upon Fe(III) complexation, and any modest apparent increase in lipophilicity does not translate into significant organic phase solubility or enhanced ability to cross lipid membranes.

CPGB exhibited 5–6-fold greater partitioning (higher OD) in octanol than DFO across all concentrations (*p* < 0.05 at all points, *p* < 0.001 at 50 and 200 µM), indicating higher hydrophobicity, while the DFO-Fe complex displayed a trend toward greater partitioning (higher OD) in octanol than the CPGB-Fe complex, with *p* < 0.05 across all points (Figure 9A). As is shown in Figure 9B, there were significant differences between the cLogP values of CPGB and DFO at all tested concentrations (50–200 µM)), while CPGB tended to show slightly higher (less negative) mean cLogP values than DFO, suggesting marginally greater hydrophobicity. In contrast, at 200 µM, the *p* value = 0.034 indicated a significant difference between DFO-Fe and CPGB-Fe complexes. The DFO-Fe complex showed a higher (less negative) mean cLogP value, suggesting it is more hydrophobic than the CPGB-Fe complex, whereas there were no significant differences at lower concentrations (50–100 µM). Therefore, there was a trend toward significance at 100 µM (*p* ≈ 0.09), which was consistent with a modest degree of divergence in partitioning behavior at higher complex concentrations.

## 3. Discussion

Iron is an indispensable element for virtually all microorganisms and is involved in sustaining cellular metabolism and proliferation. To meet this requirement, microbes have developed a variety of strategies to capture and assimilate iron from their surroundings. Among these, the synthesis of specialized siderophores via low-molecular-weight compounds (approximately 500–1500 Da) with remarkable affinity and specificity for Fe^3+^ represents one of their most prevalent and effective mechanisms. The present study has revealed that *T. marneffei* synthesizes and secretes CPGB, a hydroxamate-type siderophore, into the culture medium. In comparison, DFO, a trihydroxamate siderophore originally derived from *Streptomyces pilosus*, remains the standard therapeutic iron chelator for patients with iron overload.

The CAS assay is a well-established, universal method for detecting siderophore activity, based on the competitive chelation of Fe(III) from the dye complex, which leads to a colorimetric shift [17]. The study confirmed that *T. marneffei* secretes extracellular siderophores into the culture supernatant. A strong positive signal was observed, with activity persisting up to an 8-fold dilution and appreciable levels detectable even at a 16-fold dilution. This would underscore the high siderophore production capacity of this fungus. The intensity of the response and its persistence across serial dilutions suggest that *T. marneffei* produces siderophores at levels comparable to or exceeding those of other filamentous fungi such as *Aspergillus* and *Penicillium* species [18]. High siderophore production may reflect the organism’s ecological adaptation to iron-limited environments, as iron bioavailability is typically restricted due to its insolubility at physiological pH levels. In pathogenic fungi, siderophore production is often linked to virulence, since efficient iron acquisition provides a competitive advantage in host tissues [19]. Accordingly, *T. marneffei* is primarily an opportunistic pathogen, while its siderophore system may contribute to survival under host-imposed iron limitation. The strong positive CAS response aligns with the subsequent purification and characterization of CPGB as the major siderophore product in this system. These findings provide a functional foundation for exploring the role of CPGB, not only in fungal physiology, but also as a candidate for therapeutic iron chelation.

The purification of siderophores obtained from *T. marneffei* culture supernatants employed a sequential chromatographic strategy combining Amberlite XAD-2 resin adsorption and Sephadex LH-20 gel-permeation chromatography, followed by HPLC-DAD analysis. This multi-step approach effectively enriched fractions containing hydroxamate-type siderophores, which would be consistent with the methods used in fungal siderophore studies [20]. Fractions were evaluated using three complementary criteria: (i) CAS assay activity, (ii) spectral fingerprints, and (iii) iron-binding capacity. In the CAS assay, siderophore-positive fractions induced a decrease in OD at 630 nm, reflecting displacement of Fe(III) from the CAS dye complex [17]. Iron-binding activity was further validated by a characteristic increase in absorbance at 450 nm upon Fe(III) complexation, a hallmark spectral feature of hydroxamate siderophores [21]. In addition, the fractions exhibited strong absorbance at 230 nm, corresponding to the π–π* transitions of the hydroxamate moiety. Integration of these three readouts allowed unambiguous identification of active siderophore-containing fractions. Specifically, 230 nm (hydroxamate group) confirmed chemical identity, 430–450 nm (Fe–siderophore complex) demonstrated functional iron-binding, and 630 nm (CAS reactivity) validated biological iron competition. The UV–Vis spectrum of CPGB exhibited a strong absorption peak at 230 nm that was characteristic of the hydroxamate functional group. A secondary band at 430–450 nm, which was indicative of Fe(III)–siderophore complex formation through ligand-to-metal charge transfer (LMCT) transitions, resulted in decolorizing blue CAS-Fe(III) complex at 630 nm and iron chelation [20,22,23,24]. Pooling of positive fractions across all three assays ensured high-confidence isolation of siderophores for downstream characterization. This combinatorial workflow provided robust cross-validation, minimizing false positives that might have arisen from relying upon a single assay. Such integrative fractionation strategies are standard in natural product chemistry and have been applied broadly to siderophore isolation from fungi and bacteria [18,21]. Collectively, the data confirm that the purified product corresponds to a hydroxamate-type siderophore with strong Fe(III)-chelating capacity—later identified as CPGB. These findings provide the biochemical foundation for further kinetic, lipophilicity, and structural analyses.

Following Sephadex LH-20 fractionation, siderophore-containing fractions were subjected to comprehensive physicochemical characterization using HPLC-DAD, flash chromatography, NMR spectroscopy, HPLC-MS, and MALDI-TOF-MS. This integrative approach allowed both separation and structural confirmation of the target siderophore, CPGB, while also highlighting the presence of impurities requiring further purification. Analytical HPLC-DAD revealed two major peaks at approximately 5 and 10 min, each absorbing strongly at 230 nm, which is known to be characteristic of hydroxamate-type siderophores. This suggested the presence of structurally related compounds with distinct molecular weights but similar functional groups. Preparative HPLC and flash chromatography reproduced this separation pattern, validating the reproducibility of the two-peak distribution. Interestingly, peak alignment across methods indicated that the later-eluting HPLC fraction (10 min) corresponded to CPGB, as its retention time was consistent with a less polar siderophore species, while the earlier peak represented a structurally related but more polar compound. This polarity distinction was confirmed in flash chromatography, where solvent systems were inverted, and peak order was reversed. Such polarity-dependent retention behaviors are consistent with the physicochemical diversity of siderophore families [20]. Crude-sample NMR spectra displayed characteristic signals consistent with hydroxamate siderophores and closely resembled those of coprogen reported by Huang et al. [16]. However, spectral overlap from co-purified compounds underscored the incomplete purification and complexity of fungal metabolite mixtures. HPLC-MS provided confirmation of the analysis of CPGB, detecting a prominent molecular ion at *m*/*z* 726.5 Da, which confirmed its presence among the complex metabolite pool. Complementary MALDI-TOF-MS^−^ analyses reinforced these findings, with a major peak detected at *m*/*z* 727.5, which was consistent with CPGB. Additional experiments using diverse matrices (SA, DHB, and CHCA) yielded reproducible detection of this species, confirming the robustness of the identification. These results demonstrate the utility of employing orthogonal techniques—chromatographic, spectroscopic, and mass spectrometric—for siderophore discovery and validation. The convergent evidence confirms that CPGB is a major, but not exclusive, product of *T. marneffei* siderophore biosynthesis. The presence of multiple siderophore-related peaks raises the possibility of metabolic diversity within the fungal iron acquisition system, a feature commonly observed in filamentous fungi [18]. While CPGB was clearly identified, the low abundance (3–4% of crude extract) and background interference highlight the importance of refining purification strategies to improve yield and purity. Enhanced preparative HPLC protocols or siderophore biosynthetic pathway engineering may be required for efficient isolation.

Spectral analysis demonstrated that DFO rapidly bound Fe^3+^ upon addition to FAC, which was consistent with its well-established role as a potent and immediate chelator [25,26]. However, kinetic studies revealed distinct differences in the binding dynamics of CPGB. While CPGB did not exhibit the same instantaneous chelation as DFO, it displayed a strong, gradual, and sustained iron-binding profile, ultimately surpassing DFO in binding capacity at later time points. Previous studies have reported that DFO bound Fe(III) rapidly, almost immediately after being mixed, indicating its fast kinetics and that it is likely representative of a very high affinity constant (log K ≈ 30.6) [27] and dissociation constant (K_d_ ≈ 100 nM) [28]. Hereby, CPGB bound Fe(III) more slowly, but eventually reached a greater overall complex formation (higher OD at 450 nm after longer incubation, indicating slower kinetics, higher binding capacity, more stability, but not necessarily higher affinity). Hydroxamate-based siderophores, such as coprogen derivatives, are known to form hexadentate complexes with high stability constants, often exceeding those of synthetic chelators [20,21]. The gradual binding observed may reflect a multistep complexation process, potentially influenced by conformational changes or intermediate coordination states prior to full hexadentate saturation. The sustained chelation profile of CPGB is of particular pharmacological significance. DFO’s rapid but transient complexation is often accompanied by rapid renal clearance and a short plasma half-life, necessitating continuous infusion or repeated dosing [26]. In contrast, CPGB’s slower yet stronger binding may provide more durable iron sequestration, potentially reducing the frequency of dosing and enhancing clinical efficacy. Moreover, sustained binding may mimic the natural behavior of microbial siderophores, which are optimized for efficient iron scavenging and transport across biological membranes [29]. Overall, these results position CPGB as a promising alternative chelator with superior long-term iron-binding capacity when compared with DFO. Future studies should define the stability constants of CPGB–Fe(III) complexes, evaluate competitive binding in the presence of biologically relevant cations (e.g., Zn^2+^, Cu^2+^, Mn^2+^), and assess whether the sustained binding translates into improved therapeutic outcomes in vivo.

The physicochemical characterization of CPGB highlights key differences when compared with the clinically used DFO. Partitioning assays revealed that CPGB exhibits moderate hydrophilicity, whereas DFO remains strongly hydrophilic. The partition values indicate that CPGB is significantly more lipophilic than DFO, suggesting greater potential for passive membrane diffusion. This difference is of pharmacological importance, as lipophilicity often correlates with improved bioavailability and tissue penetration of chelators [30]. Upon complexation with Fe(III), both siderophores showed reduced lipophilicity, which was consistent with previous reports that metal binding introduces additional polarity and hydration shells [31]. However, CPGB–Fe(III) retained moderate hydrophobicity, with ~30–35% partitioning into the octanol phase, while DFO–Fe (FO) remained almost exclusively in the aqueous phase (~7–8%). This suggests that CPGB–Fe complexes may mimic bacterial siderophores designed for membrane-associated transport, potentially enabling superior transmembrane trafficking and distribution in vivo [20,29]. In contrast, the bulky, charged FO complex of DFO is highly aqueous, limiting its biodistribution and contributing to its known short plasma half-life and requirement for parenteral administration [32]. The moderate lipophilicity of CPGB aligns with the desirable characteristics for orally bioavailable chelators. Existing alternatives, such as DFX and DFP, were developed partly due to DFO’s pharmacokinetic drawbacks, but each carries unique toxicity liabilities [26,33]. CPGB’s balance of aqueous solubility and partial hydrophobicity may allow efficient systemic distribution while retaining sufficient clearance capacity. Furthermore, its sustained Fe(III)-binding activity, comparable or superior to DFO, positions it as a strong candidate for therapeutic development.

In terms of an advantage, cooperative interactions between siderophores and conventional antibiotics have been shown to suppress the proliferation of highly drug-resistant pathogens, including methicillin-resistant *Staphylococcus aureus*, metallo-β-lactamase-producing *Pseudomonas aeruginosa*, and *Acinetobacter baumannii*. The current study establishes a strong biochemical foundation for CPGB characterization but requires further purification, stability studies, selectivity testing, and in vivo validation before therapeutic potential can be conclusively determined.

## 4. Materials and Methods

### 4.1. Reagents and Chemicals

All reagents and chemicals used in this study were obtained from commercial suppliers. Specifically, MOPS, CAS, FAC, ammonium sulfate, uracil, sodium chloride, and Tween-40 were purchased from Sigma-Aldrich Chemicals Company Limited (Saint Louis, MO, USA). Aspergillus Nitrogen-Free Medium (ANM) was acquired from Gibco, a division of Thermo Fisher Scientific. DFO was obtained from a drug store in Maharaj Nakorn Chiang Mai Hospital, Faculty of Medicine, Chiang Mai University, Chiang Mai, Thailand. Amberlite XAD-2 (Product number 10357, 20–60 mesh particle size, 90 Å mean pore size) and Sephadex LH-20 (Product number LH20100, bead size 25–100 μm) resin were bought from the Sigma-Aldrich Company. Acetonitrile, formic acid, hydrochloric acid, methanol, and TFA were all HPLC- or Anala-grade. The mutant strain *ΔsreA* was kindly provided to us by Dr. Monsicha Pongpom from the Department of Microbiology, Faculty of Medicine, Chiang Mai University, Thailand.

### 4.2. Culture and Germination of Talaromyces marneffei sre Mutant Strain

This research study utilized genetically modified *ΔsreA* of *T. marneffei*, which was cultivated on ANM supplemented with 10 mM ammonium sulfate as a nitrogen source and 5 mM uracil to enhance fungal growth. The *ΔsreA* strain was cultured on uracil-free ANM. All strains were grown on ANM for at least 10 days. Conidia were collected from mature mycelial colonies grown on ANM plates at room temperature for 10–14 days. To harvest conidia, plates were gently flooded with 10 mL of sterile saline–Tween solution (0.85% NaCl, 0.1% Tween-40), and spores were dislodged by lightly scraping plates with a sterile L-shaped spreader purchased from Thermo Fisher Scientific (Waltham, MA, USA). The resulting suspension was filtered through sterile glass wool inside a syringe barrel to eliminate hyphal debris, yielding a purified conidial suspension. Conidia were washed three times by centrifugation at 5000× *g* for 10 min, with each pellet resuspended in sterile saline. The final spore concentration was measured using a hemocytometer under a light microscope. The conidia were prepared to a final concentration of 1 × 10^6^ conidia/mL in ANM broth. The culture filtrate was collected from a 7-day culture for further purification, as described below.

### 4.3. Extracellular Siderophore Purification

Extracellular siderophores secreted by the *ΔsreA* mutant were recovered from culture supernatants for purification. The fungus was cultivated in a 5 L culture system, with inoculation into ANM broth at a final density of 1 × 10^6^ conidia/mL. Cultures were maintained at room temperature under constant agitation at 200 rpm for seven days. After incubation, cells were removed by centrifugation, and the supernatant containing siderophores was collected and passed through a filtration membrane (Cellulose type, 0.45 µm pore size) to eliminate residual particulates [13].

### 4.4. Amberlite XAD and Sephadex LH-20 Column Chromatography

Purification of siderophores began with column chromatography using Amberlite XAD-2 and Sephadex LH-20 beads [34]. Amberlite XAD-2 columns separate molecules based on both hydrophobicity and size, while Sephadex LH-20 primarily separates them by molecular weight. Fifty grams of Amberlite XAD-2 beads was pre-soaked in methanol for one hour, rinsed thoroughly with distilled water, treated with pH 2.0 HCl, and then packed carefully to avoid gaps or air bubbles. Columns were subsequently washed with 500 mL of deionized water twice. The culture supernatant was acidified to pH 2 with 6 M HCl before being loaded onto the water-equilibrated Amberlite XAD-2 column. Bound siderophores were eluted with methanol, yielding 90 fractions of 5 mL each, which were screened using CAS assays, iron-binding assays, and UV-Vis spectroscopy (200–700 nm). Fractions testing positive were pooled, concentrated using a rotary evaporator, and freeze-dried into powder. The dried material was dissolved in methanol and subjected to Sephadex LH-20 chromatography to refine separation by molecular size and hydrophobicity. Positive fractions were further purified by reverse-phase (RP)-HPLC on an Agilent Zorbax SB-C18 column (Part number 50648262, 80 Å, 150 mm × 0.5 mm, 3.5 µm particle size, Agilent Technologies Inc., Santa Clara, CA, USA) using two mobile phases: solvent A (0.1% formic acid in water) and solvent B (0.1% formic acid in acetonitrile). Purified siderophores were collected, concentrated, re-dissolved in deionized water, and stored as freeze-dried powder at −4 °C for later use.

### 4.5. Identification of Positive Fraction

The fractions that were positive after Amberlite XAD-2 chromatography were combined and subjected to Sephadex LH-20 chromatography, while those that were positive after Sephadex LH-20 chromatography were pooled and analyzed by high-performance liquid chromatography-diode array detection (HPLC-DAD). In the CAS assay, positive fractions exhibited a reduction in optical density (OD) at 630 nm, whereas those with iron-binding capacity showed an increase in A at 450 nm. When integrated with spectral data at 230 nm, we were able to identify the fractions that were positive for siderophores.

#### 4.5.1. Spectrophotometric Analysis

Each chemical species has a characteristic absorption spectrum that helps in identifying its nature. Hydroxamate-type siderophores typically absorb light between 210 and 250 nm, with a distinctive peak around 230 nm. When complexed with iron, these molecules exhibited dominant OD values between 430 and 450 nm [35]. The Beer–Lambert law allows estimation of their concentration in solution based on A values. For analysis, pooled fractions were diluted in methanol at a 1:10 ratio (100 µL fraction + 900 µL methanol). Using 1 mL quartz cuvettes, fractions collected from chromatography were scanned across 200–700 nm to confirm siderophore presence and assess possible iron contamination.

#### 4.5.2. Colorimetric Iron-Binding Assay

Fractions obtained from column chromatography were tested for iron-binding ability using FAC reagent [35]. In a 96-well plate, 100 µL of each fraction was mixed with 100 µL of FAC solution, and OD was measured at 450 nm at different time intervals (0, 15, 30, 60, 90, and 120 min) using a double-beam UV-Vis spectrophotometer (BioTek™ Synergy™ H4 Hybrid Reader, Winooski, VT, USA). Fractions showing a marked absorbance peak at 450 nm after 30 min were identified as siderophore-positive, since this wavelength shift indicated the formation of siderophore–iron complexes.

#### 4.5.3. CAS Assay

To evaluate siderophore activity using the CAS assay, 100 µL of each chromatographic fraction was combined with 100 µL of CAS reagent in a 96-well plate. A value of 630 nm was recorded at 0, 15, 30, 60, 90, and 120 min [13]. Fractions that caused a significant reduction in OD units at 630 nm relative to the methanol blank were identified as positive, confirming their ability to alter the CAS reagent through iron chelation.

### 4.6. Purification and Charecterization of Hydroxamate Siderophore

#### 4.6.1. Analytical HPLC Analysis

Further purification of siderophores was confirmed using reverse-phase HPLC on an Agilent Zorbax SB-C18 column (dimension 150 mm x 3.0 mm, 5-μm particle size, Agilent Technologies Inc., Santa Clara, CA, USA). The mobile phases consisted of solvent A (0.1% formic acid in DI water) and solvent B (0.1% formic acid in acetonitrile). The gradient program was as follows 0 min, 100% A; 20 min, 10% A/90% B; 25 min, 100% A/0% B; total run time, 30 min. Injections of 10 µL were applied at a flow rate of 0.5–1 mL/minute, with the column maintained at 40 °C. Accordingly, OD was monitored at 230, 254, and 450 nm. Fractions showing the most prominent chromatographic peaks were compared with published reference data to determine the siderophore type.

#### 4.6.2. Preparative HPLC Analysis

Further purification of siderophores was confirmed using reverse-phase HPLC on an Infinity Zorbax Eclipse Plus C18 column (dimension 250 mm × 21.2 mm, 5-μm particle size, Agilent Technologies Inc., Santa Clara, CA, USA). The mobile phases consisted of solvent A (0.1% formic acid in water) and solvent B (0.1% formic acid in acetonitrile). The gradient program was as follows: 0 min, 100% A; 20 min, 10% A/90% B; 25 min, 100% A/0% B; total run time, 30 min. Injections of 212 µL were applied at a flow rate of 17–34 mL/minute, with the column maintained at 40 °C. Accordingly, OD was monitored at 230, 254, and 450 nm. Fractions showing the most prominent chromatographic peaks were compared with published reference data to determine the siderophore type.

#### 4.6.3. Flash Chromatography

Flash chromatography was employed as a rapid purification strategy for organic molecules, natural products, and peptides. Unlike conventional gravity-based chromatography, this method uses pressurized gas (50–200 psi) to accelerate separation, making it more efficient and capable of handling larger sample volumes than standard HPLC columns. In this study, a Shim-pack PREP-ODS column (Shimadzu Corporation, Kyoto, Japan, 20 mm × 250 mm, 10 μm particle size) was used in a Flash Chromatography system (Model: Pure C-815, Lab Pilot Process Equipment AG, Uster, Switzerland) with acetonitrile (A) and water (B) as mobile phases. The solvent gradient was programmed as follows: 0 min, 99% A/1% B; 24 column volumes, 50% A/50% B; total run time, 30 min. The flow rate was maintained at 28 mL/minute, and UV detection was set at 210, 220, 230, and 450 nm. Fractions corresponding to major peaks were collected for further characterization.

#### 4.6.4. HPLC-MS Analysis

HPLC-MS technique was used to chemically profile the siderophores secreted by the *ΔsreA* strain of *T. marneffei* [34]. The HPLC system (Agilent Technologies 1100 Series, Deutschland GmbH, Waldbronn, Germany) consisted of a quaternary pump (G1311A), an online vacuum degasser (G1322A), an autosampler (G1313A), a thermally regulated column compartment (G1316A), and a PDA detector (G1315A) that utilized a Symmetry^®^ C18 column (4.6 mm × 100 mm, 5 µm; Milford, MA, USA) with solvent A (0.1% formic acid in water) and solvent B (0.1% formic acid in acetonitrile). The gradient was programmed as follows: 0 min, 98% A/2% B; 11 min, 5% A/95% B; 11.1–15 min, 98% A/2% B; total run time, 30 min. A 5 µL injection volume was applied with a flow rate of 0.5 mL/minute at 37 °C. The eluted compounds were introduced into an Agilent 6490 Triple Quadrupole mass spectrometer (Agilent Technologies 1100 LC/MSD SL, Palo Alto, CA, USA) through a flow splitter (1:1) operating in negative electrospray ionization (ESI^−^) mode. Molecular ions were then analyzed according to their *m*/*z* ratios.

#### 4.6.5. MALDI-TOF/TOF-MS Analysis

Herein, MALDI-TOF-MS was employed to analyze the siderophores, a method widely used for biomolecules such as proteins, nucleic acids, peptides, saccharides, and large polymers. In this technique, samples are embedded in a crystalline matrix and ionized by laser irradiation, allowing them to vaporize into the gas phase without decomposition. For this study, 1 µg of the siderophore was dissolved in 1 mL DI water. One microliter of different matrices (SA, CHCA, and DHB), spotted in duplicate, was dried on a MALDI plate before being overlaid with 1 µL of siderophore solution. Analyses were performed using the Shimadzu Performance iD Plus MALDI-TOF-MS system (Shimadzu Corporation, Kyoto, Japan) to generate mass spectra.

#### 4.6.6. NMR Spectroscopy Analysis

NMR spectroscopy was performed to elucidate the molecular structure of extracellular siderophores produced by *T. marneffei* [34]. Both crude and purified samples were dissolved in 0.5 mL of deuterium oxide (D_2_O). Proton NMR (^1^H NMR) spectra were recorded on a Bruker NEO™ 500 MHz instrument (AVANCE NEO500, Bruker BioSpin, Zurich, Switzerland) at room temperature with an internal deuterium lock. Chemical shifts (δ) were reported in parts per million (ppm) to the nearest 0.01 ppm and referenced against residual solvent peaks. The obtained spectra were then compared with published data for structural interpretation.

### 4.7. Investigation of Siderophore Activities

#### 4.7.1. Spectral Analysis

Spectrophotometric scanning (200–700 nm) of the purified siderophore was conducted to confirm whether it belonged to the hydroxamate class [36]. This basic method also provided insight into complex formation and structural features. Samples were prepared at concentrations of 12.5, 25, 50, and 100 µM in 100 µM MOPS buffer pH 7.4. Measurements of OD values were taken in quartz cuvettes using a double-beam UV-Vis spectrophotometer (BioTek™ Synergy™ H4 Hybrid Reader, Vermont, USA), with MOPS buffer serving as the blank control.

#### 4.7.2. Kinetics of Iron Binding to Iron

To investigate iron-binding properties, siderophore samples at concentrations of 12.5, 50, 100, and 200 µM were incubated with 500 µM FAC prepared in 100 µM MOPS buffer pH 7.4. Following 15 min of incubation, the siderophore–iron complexes were analyzed over the 200–700 nm spectral range using a scanning double-beam UV-Vis spectrophotometer (BioTek™ Synergy™ H4 Hybrid Reader). MOPS buffer was used as a blank [35]. The appearance of a distinct OD within 350–550 nm indicated the formation of ferric hydroxamate siderophore complexes.

##### Time-Couse Effect

To evaluate the kinetics of iron binding, siderophore solutions at concentrations of 32.5, 75, 150, 300, and 600 µM were mixed with 300 µM FAC prepared in 100 µM MOPS buffer. The formation of siderophore–iron complexes was monitored by measuring absorbance at 450 nm, a wavelength characteristic of such complexes, at time intervals of 15, 30, 60, 90, and 120 min using a scanning double-beam UV-Vis spectrophotometer (BioTek™ Synergy™ H4 Hybrid Reader). In a complementary assay, the experimental setup was inverted as follows: FAC was prepared at concentrations of 32.5, 75, 150, 300, and 600 µM and incubated with a fixed concentration of 300 µM siderophore in 100 µM MOPS buffer pH 7.4 [32]. Accordingly, an OD value of 450 nm was again recorded at the same time intervals to further characterize the dynamics of complex formation.

##### Dose–Response Effect

Iron mobilization capacity was assessed by testing siderophores against oligomeric iron(III) citrate using visible spectrophotometry. A 100 µM iron–citrate solution (molar ratio Fe:citrate = 1:10) was prepared in 100 mM MOPS buffer at pH 7.4 [37]. This solution was mixed with the siderophore at concentrations of 32.5, 75, 150, 300, and 600 µM, along with equivalent concentrations of the reference chelator DFO. Accordingly, an OD value of 450 nm was measured every 30 s over a 4 min period to monitor iron release kinetics from the citrate complex.

### 4.8. Determination of Partition Coefficient

Partitioning refers to how a compound distributes itself between two immiscible solvents, water/*n*-butanol or water/*n*-octanol. It was measured by comparing the A values of CPGB and DFO itself at 230 nm and those of CPGB-Fe(III) and DFO-Fe(III) complexes at 450 nm, before and after the addition of *n*-octanol. Accordingly, the ratio of A values in *n*-octanol versus aqueous buffer gave the partition coefficient (*p* or K_part_) value. This meant that the higher partitioning into *n*-octanol, the more lipophilic the compound was, while the higher partitioning into water, the more hydrophilic the compound was. Thus, the partitioning of CPGB and DFO siderophores was determined using both *n*-octanol/water biphasic systems [35]. In each assay, 2.5 mL of *n*-octanol was added to 2.5 mL of 50 mM MOPS buffer (pH 7.4) containing CPGB and DFO (50–200 µM), and CPGB-Fe(III) and DFO-Fe(III) (25–200 µM). The mixture was stirred for 45 min, left to equilibrate for 15 min, and then centrifuged. Afterwards, 1.0 mL of the aqueous phase was withdrawn, and OD values were measured before and after solvent extraction. Mathematically,

(1) $$P=\frac{\left({OD}_{1}-{OD}_{2}\right)}{{OD}_{2}}\times \frac{V_{w}}{V_{o}}$$

where *OD*_1_ and *OD*_2_ represent OD_230_ values (for siderophores) and OD_450_ values (for complex) of the aqueous phase before and after solvent extraction, respectively, and *V_w_* and *V_o_* correspond to the volumes of the aqueous and organic phases. cLogP is the calculated (predicted) logarithm *p* value between *n*-octanol and water using the following formula:

cLogP = log10 (OD_octanol_/OD_water_) (2)

A high cLogP value means the compound is hydrophobic (or lipophilic); inversely, a low or negative cLogP means the compound is hydrophilic.

### 4.9. Data and Statistical Analysis

All quantitative experiments were performed in biological triplicate (n = 3) unless otherwise specified. Data were analyzed using the Statistical Package for the Social Sciences (SPSS) Statistics for Windows version 22 Program (IBM Corporation, Armonk, NY, USA) and expressed as values of mean ± standard deviation (SD).

## 5. Conclusions

This study employed powerful chromatographic and spectroscopic methods for the purification of the hexadentate siderophore CPGB obtained from the *ΔsreA* mutant strain of *Talaromyces marneffei*. The systematic separation of the CPGB siderophore presented here holds considerable potential for its translational applications. Following purification, CPGB was employed to compare its iron-chelating and iron-binding capacity to that of DFO. However, it was found in some instances to be superior to DFO. In addition, free CPGB and the CPGB–iron complex displayed reduced lipophilicity when compared with free DFO and the DFO–iron complex, suggesting the potential for more efficient excretion. Future studies should focus on pharmacokinetics, metabolic stability, and safety in animal models. Particularly, the evaluation of oral absorption, tissue distribution, and the inclusion of potential off-target interactions would be critical in validating whether CPGB’s physicochemical advantages translate into clinical benefits. Additionally, structural derivatization or formulation strategies could further optimize its therapeutic index.

## Data Availability

All data presented in this study are available from the corresponding author upon reasonable request.

## Abbreviations

The following abbreviations are used in this manuscript:

ANM	*Aspergillus* nitrogen-free medium
CAS	Chrome azurol S
CHCA	α-Cyano-4-hydroxycinnamic acid
cLogP	Computer-predicted log P
CPGB	Coprogen B
DAD	Diode array detector
DFO	Deferoxamine
DFP	Deferiprone
DFX	Deferasirox
DHB	2,5-Dihydroxybenzoic acid
DI	Deionized
D_2_O	Deuterium oxide
ESI	Electrospray ionization
FAB	Fast atom bombard
FAC	Ferric ammonium citrate
Fe^2+^	Ferrous ion
Fe^3+^	Ferric ion
FO	Ferrioxamine
FT-IR	Fourier-Transform infrared
Ga	Gallium
MALDI	Matrix-assisted laser desorption/ionization
MOPS	3-(N-Morpholino)propanesulfonic acid
MS	Mass spectrometry
MW	Molecular weight
*m*/*z*	Mass-to-charge ratio
ND	Not determined
NMR	Nuclear magnetic resonance
OD	Optical density
P	Partition, partitioning
ppm	Parts per million
QTOF	Quadrupole time-of-flight
RP	Reverse phase
SA	Sinapinic acid
SD	Standard deviation
SreA	Siderophore-regulatory element
*T. marneffei*	*Talaromyces marneffei*
TFA	Trifluoroacetic acid
T_R_	Retention time
UV/Vis	Ultraviolet/visible
XRD	X-ray diffraction
